# Radiomics-based machine learning models for differentiating pathological subtypes in cervical cancer: a multicenter study

**DOI:** 10.3389/fonc.2024.1346336

**Published:** 2024-09-17

**Authors:** Huiling Liu, Mi Lao, Yalin Zhang, Cheng Chang, Yong Yin, Ruozheng Wang

**Affiliations:** ^1^ Department of Radiation Oncology, The Third Affiliated Teaching Hospital of Xinjiang Medical University, Affiliated Cancer Hospital, Urumuqi, China; ^2^ Department of Radiation Oncology, Binzhou People’s Hospital, Binzhou, China; ^3^ Department of Cardiology, Binzhou People’s Hospital, Binzhou, China; ^4^ Department of Nuclear Medicine, The Third Affiliated Teaching Hospital of Xinjiang Medical University, Affiliated Cancer Hospital, Urumuqi, China; ^5^ Department of Radiation Oncology, Shandong Cancer Hospital and Institute, Shandong First Medical University and Shandong Academy of Medical Sciences, Jinan, China; ^6^ Key Laboratory of Oncology of Xinjiang Uyghur Autonomous Region, Urumuqi, China; ^7^ Clinical Key Specialty of Radiotherapy of Xinjiang Uygur Autonomous Region, Urumuqi, China

**Keywords:** locally advanced cervical cancer, positron emission tomography, PET, radiomics, adenocarcinoma, AC, squamous cell carcinoma, SCC

## Abstract

**Purpose:**

This study was designed to determine the diagnostic performance of fluorine-18-fluorodeoxyglucose (^18^F-FDG) positron emission tomography (PET)/computed tomography (CT) radiomics-based machine learning (ML) in the classification of cervical adenocarcinoma (AC) and squamous cell carcinoma (SCC).

**Methods:**

Pretreatment ^18^F-FDG PET/CT data were retrospectively collected from patients who were diagnosed with locally advanced cervical cancer at two centers. Radiomics features were extracted and selected by the Pearson correlation coefficient and least absolute shrinkage and selection operator regression analysis. Six ML algorithms were then applied to establish models, and the best-performing classifier was selected based on accuracy, sensitivity, specificity, and area under the curve (AUC). The performance of different model was assessed and compared using the DeLong test.

**Results:**

A total of 227 patients with locally advanced cervical cancer were enrolled in this study (N=136 for the training cohort, N=59 for the internal validation cohort, and N=32 for the external validation cohort). The PET radiomics model constructed based on the lightGBM algorithm had an accuracy of 0.915 and an AUC of 0.851 (95% confidence interval [CI], 0.715-0.986) in the internal validation cohort, which were higher than those of the CT radiomics model (accuracy: 0.661; AUC: 0.513 [95% CI, 0.339-0.688]). The DeLong test revealed no significant difference in AUC between the combined radiomics model and the PET radiomics model in either the training cohort (*z*=0.940, P=0.347) or the internal validation cohort (*z*=0.285, P=0.776). In the external validation cohort, the lightGBM-based PET radiomics model achieved good discrimination between SCC and AC (AUC = 0.730).

**Conclusions:**

The lightGBM-based PET radiomics model had great potential to predict the fine histological subtypes of locally advanced cervical cancer and might serve as a promising noninvasive approach for the diagnosis and management of locally advanced cervical cancer.

## 1 Introduction

Cervical cancer is the fourth most common female cancer worldwide (1). In 2016, there were approximately 34,000 cervical cancer-related deaths in Chinese women (2). Squamous cell carcinoma (SCC) and adenocarcinoma (AC) are the main pathological subtypes of cervical cancer, accounting for 70-75% and 10-25% respectively. The incidence of AC has been observed to increase in recent decades (3). Patients with locally advanced cervical cancer who receive radiation therapy or concurrent chemoradiotherapy have a worse prognosis for AC compared to SCC, highlighting the need for alternative treatment options specifically for AC cases (4). High intratumor heterogeneity exhibited a significantly poor clinical outcome (5). Therefore, it is essential to uncover the differences between AC and SCC from multiple perspectives, explore the underlying reasons for these differences, and develop personalized treatment strategies and plans, as this holds considerable importance.

Pathological diagnosis is considered the gold standard for the detection of cervical cancer, with cervical cytology and cervical biopsy being the primary recommended methods (6). However, AC may sometimes result in cytological false-negatives (7). Biopsy is an invasive procedure associated with risks of bleeding and infection. Point-to-point biopsy performed on larger tumors only evaluates a small portion of the sample, resulting in sampling bias and an inability to comprehensively assess tumor heterogeneity (8–10). In addition, radiologists find it challenging to differentiate AC from SCC based on conventional imaging modalities such as magnetic resonance imaging (MRI), positron emission tomography (PET)/computed tomography (CT), ultrasound, etc., and the interobserver agreement is typically low (11, 12).

Radiomics is a rapidly growing field of research that utilizes medical images to extract quantitative features, converting them into high-dimensional data for analysis and exploration. This technique enhances our understanding of diseases and provides valuable support for clinical decision-making (13, 14). Malignant tumors exhibit considerable spatial variation within the tumor at the morphological and histopathological levels, including cellularity, vascularization, extracellular matrix, and necrotic components (15, 16). As a noninvasive tool, radiomics can quantify intratumoral heterogeneity and is widely used in diagnosis, treatment response evaluation, and survival prediction (17). Among conventional imaging modalities, MRI has better fine exquisite soft tissue resolution than CT and PET, so it has long been considered as the preferred imaging method of choice for the evaluation of local tumor extension in primary cervical cancer. Unsurprisingly, numerous radiomics studies aimed at identifying the pathological subtypes of cervical cancer are primarily founded on pretreatment MRI (18). To the best of our knowledge, there are no radiomics studies that delineate the primary cervical tumor on CT images, which might be related to the inability to clearly define the boundary of the primary cervical tumor on CT images. In addition, only two published studies have preliminarily evaluated PET radiomics in cervical cancer to discriminate between AC and SCC (19, 20). Nevertheless, both of the studies were single-center ones, extracted too few radiomic features, and the methods for radiomics feature selection and model construction were simplistic. Even in one of the studies, only 83 patients were included.

Previous studies have shown that radiomic features based on CT or PET images can achieve the differentiation of pathological types of lung cancer (21). The application of radiomics methods for diagnosis and tumor characterization might be a potential supplement for omics datasets, or an alternative for pathological diagnosis, particularly for patients who are at an advanced stage, inoperable, or unable to undergo biopsies. A more extensive and comprehensive study is required to investigate the value of PET/CT imaging in differentiating the subtypes of cervical cancer. Therefore, the aim of this study was to develop and validate an optimal machine learning (ML) model based on pretherapeutic fluorine-18-fluorodeoxyglucose (^18^F-FDG) PET/CT for differentiating between SCC and AC in cervical cancer.

## 2 Materials and methods

### 2.1 Study design

**Figure 1:**
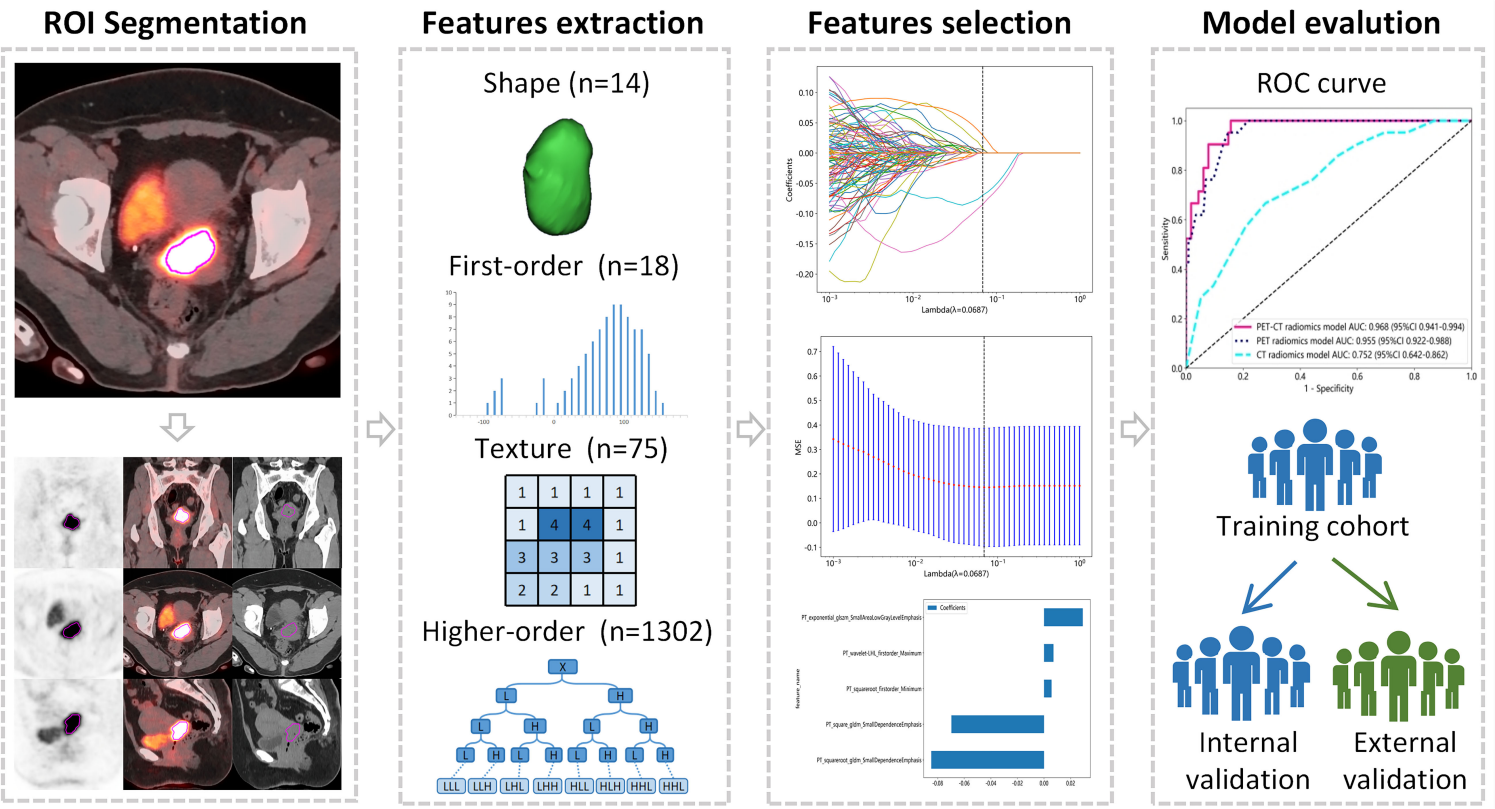
The Workflow of this study.

This retrospective study was conducted in accordance with the Declaration of Helsinki. Ethical approval was obtained from the Institutional Review Board of the Affiliated Cancer Hospital of Shandong First Medical University (No. SDTHEC2023006030) and the Affiliated Cancer Hospital of Xinjiang Medical University (No. K-2022006), and the requirement for written informed consent was waived. The workflow of our study is shown in **Figure 1**.

### 2.2 Patient cohort

The study included patients with a diagnosis of cervical cancer between September 2015 and February 2022. The inclusion criteria were as follows: (1) pathologically confirmed cervical cancer with the 2018 International Federation of Gynecology and Obstetrics (FIGO) stage IB-IVA; (2) underwent ^18^F-FDG PET/CT; and (3) complete clinical data retrievable from the electronic medical records. Exclusion criteria included: (1) a history of any previous anticancer treatment; (2) pathological types other than SCC and AC; (3) patients with a diagnosis of other unrelated malignant tumors; (4) presence of extensive abdominal metastasis; (5) poor PET/CT image quality; and (6) primary maximal tumor diameter less than 1.0 cm.

**Figure 2:**
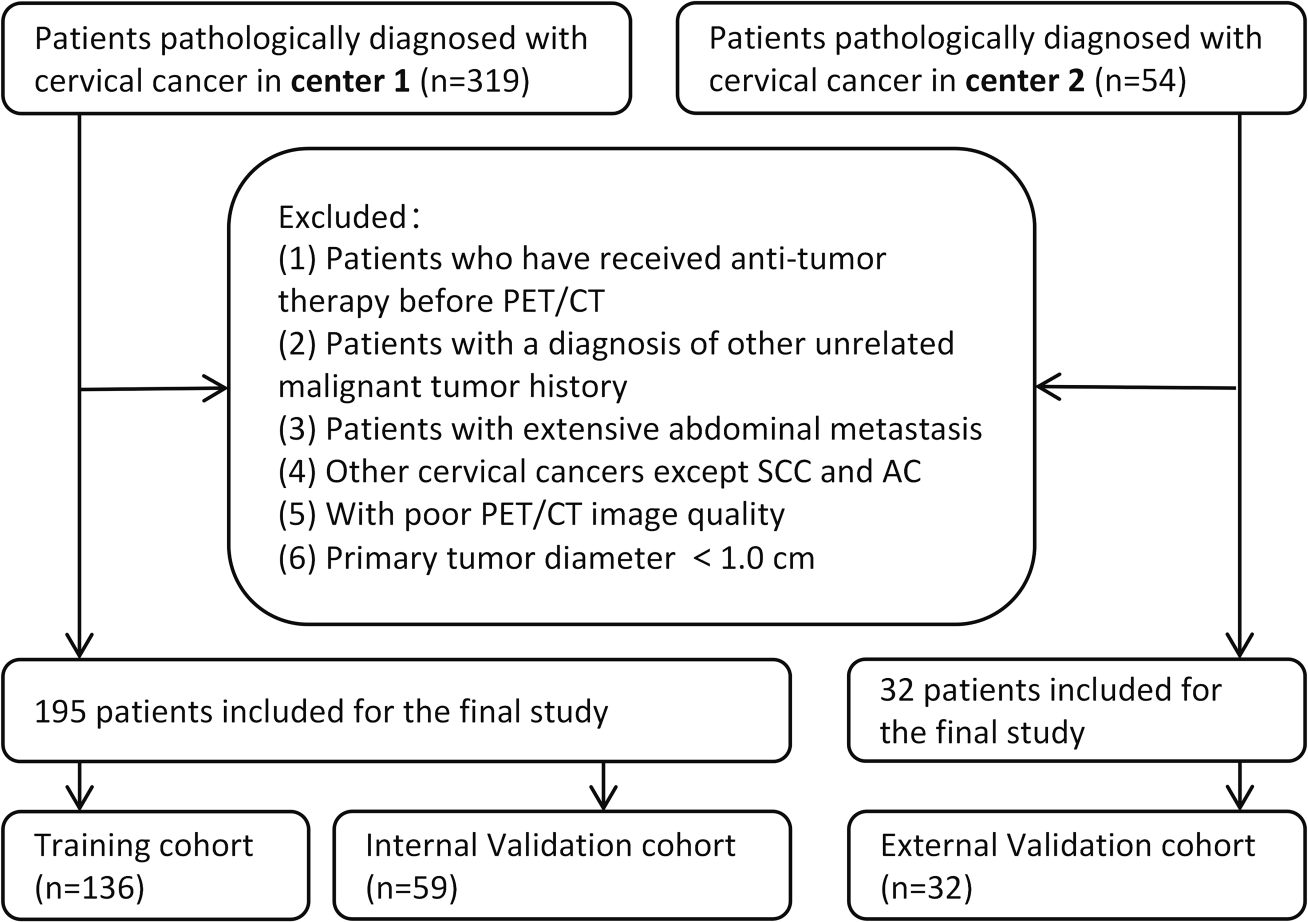
Flow chart of patients selection.

All patients were initially confirmed by hematoxylin-eosin (HE) staining, and the poorly differentiated patients whose subtypes could not be affirmed were further confirmed by immunohistochemistry (IHC) staining. Ultimately, based on the pathological reports of biopsy specimens, a total of 195 patients were recruited in the Center 1 (the Affiliated Cancer Hospital of Shandong First Medical University), among which 164 were confirmed by HE staining and 31 were confirmed by IHC. The Center 2 (the Affiliated Cancer Hospital of Xinjiang Medical University) recruited 32 patients, of whom 23 were confirmed by HE staining and 9 were confirmed by IHC. The patients recruited in the Center 1 were randomly allocated to the training cohort (n = 136) and the internal validation cohort (n = 59) in a 7:3 ratio, while the Center 2 serves as the external validation cohort. **Figure 2** illustrates a flow chart outlining the process of patient selection. The clinical information of the patients, including age, pathology, maximal tumor diameter (MTD) on PET/CT images, menopausal status, lymph node metastasis (LNM), and red blood cell count, was collected from electronic medical records.

### 2.3 PET/CT acquisition

All enrolled patients underwent ^18^F-FDG PET/CT with a standardized scan setup and parameters before treatment. Patients enrolled in Center 1 were scanned with the Philips Gemini TF PET/CT scanner (Phillips Medical Systems, Holland), and the ^18^F-FDG was generated by the MINItrace cyclotron from GE Healthcare. At Center 2, the Philips Ingenuity TF (Phillips Medical Systems, Holland) was used, and ^18^F-FDG was generated by the Sumitomo Heavy Industries HM-10 cyclotron. The radiochemical purity was above 95%. All patients fasted for at least 6 h, and their peripheral blood glucose levels were confirmed to be ≤150 mg/dL before ^18^F-FDG injection. ^18^F-FDG was intravenously administered at 3.7–4.4 MBq/kg body weight. The key scanning parameters were as follows: tube voltage of 120-130 KV; tube current of 150-300 mA. PET images were reconstructed using ordered-subset expectation maximization. Reconstruction using standard convolution kernel with 1.5 mm layer thickness (median 1.5 mm; range 1.0–3.0 mm). Each CT image was reconstructed in a 512×512 pixels image matrix and each PET image was reconstructed in a 144×144 pixel image matrix. To eliminate image differences between images acquired by different scanners, all images were resampled to the same image spacing of 1 mm×1 mm ×1 mm.

### 2.4 Tumor segmentation

**Figure 3:**
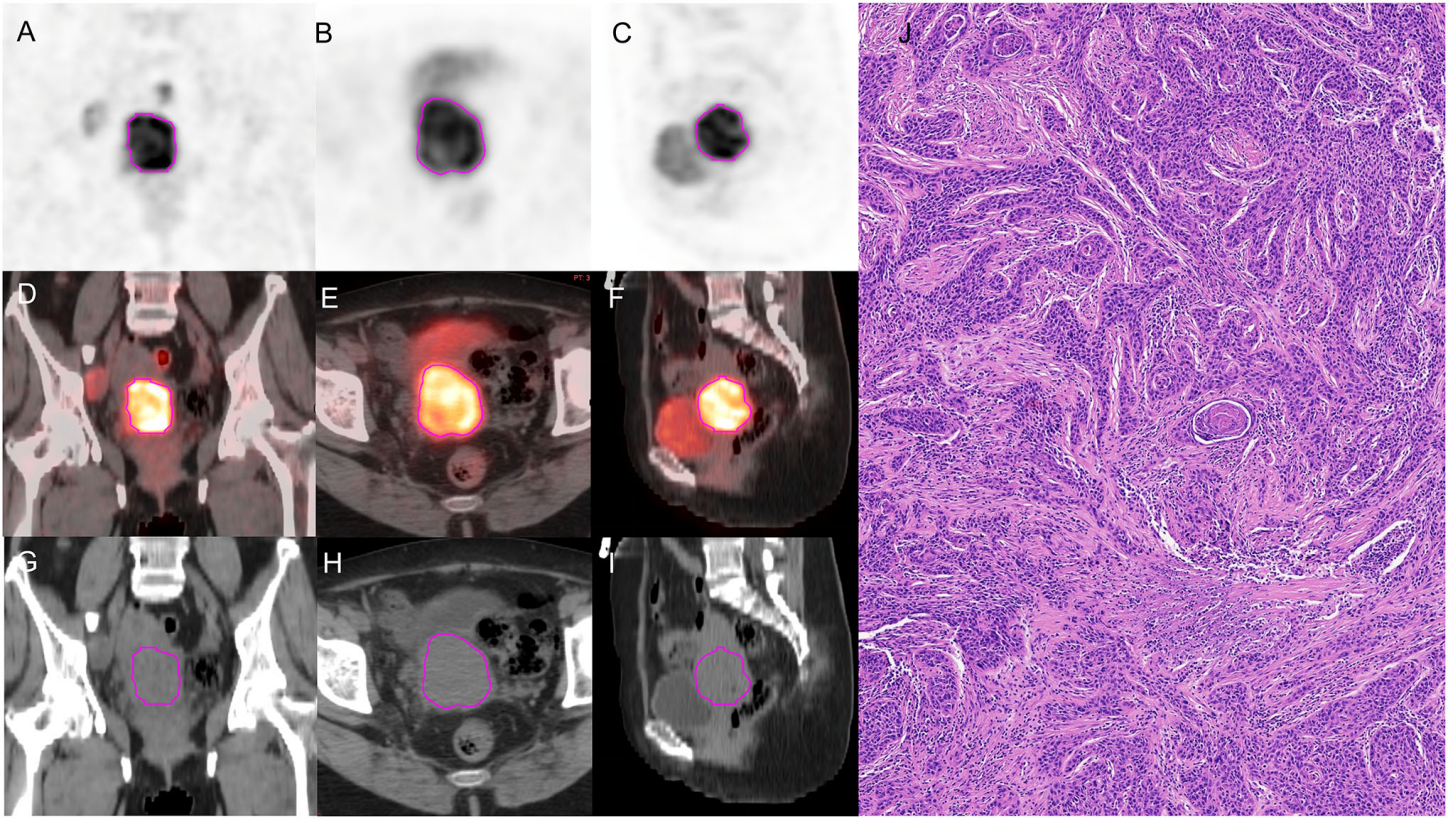
A 53-year-old woman diagnosed with SCC. **(A–C)** The ROI, labeled in red, was segmented on the coronal, axial, sagittal PET images. **(D–F)** The ROI, labeled in red, was segmented on the coronal, axial, sagittal fusion images. **(G–I)** The ROI, labeled in red, was segmented on the coronal, axial, sagittal CT images. **(J)** pathological examination confirmed SCC.

**Figure 4:**
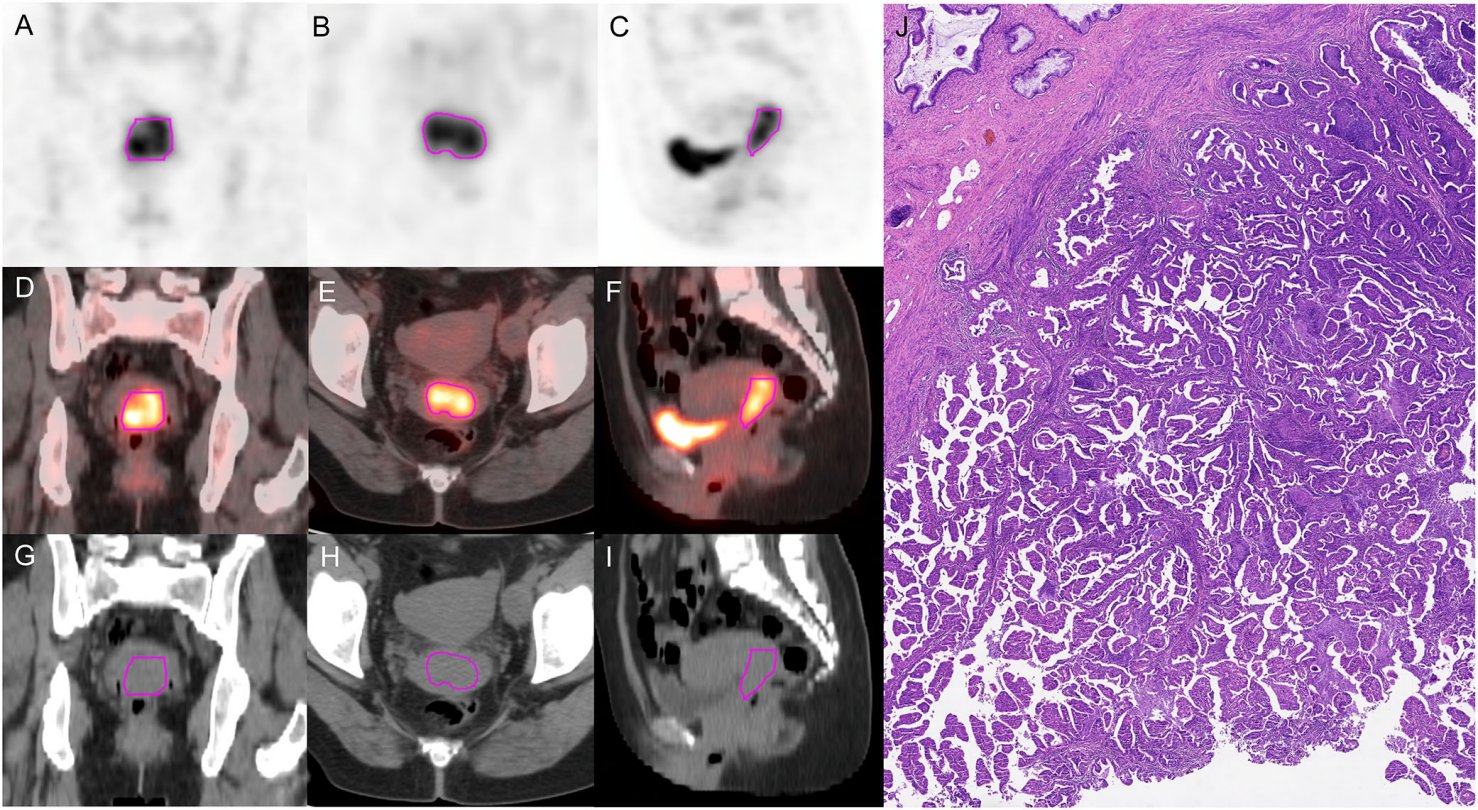
A 41-year-old woman diagnosed with AC. **(A–C)** The ROI, labeled in red, was segmented on the coronal, axial, sagittal PET images. **(D–F)** The ROI, labeled in red, was segmented on the coronal, axial, sagittal fusion images. **(G–I)** The ROI, labeled in red, was segmented on the coronal, axial, sagittal CT images. **(J)** Pathological examination confirmed AC.

PET images were attenuated, corrected, reconstructed in multiple layers, and then fused with noncontrast-enhanced low-dose CT images. The resulting images were imported into MIM Maestro version 7.1.7 (MIM Software Inc., Cleveland, OH, USA). The regions of interest (ROIs) were delineated using a fixed threshold value at 42% of the maximum standardized uptake value (SUVmax) of the primary tumor. Regions corresponding to the bladder were manually excluded from the analysis. For the obtained ROIs, various parameters, such as metabolic active tumor volume (MTV), mean standardized uptake value (SUVmean), total lesion glycolysis (TLG), and SUVmax, were calculated using MIM Software. The contoured ROIs were then transferred to PET and CT images using rigid registration. Another experienced oncologist carefully reviewed and modified the transferred results on a slice-by-slice basis. **Figures 3**, **4** show a set of representative PET/CT images from a 53-year-old woman with SCC and a 41-year-old woman with AC, respectively. The ROI, labeled in red, was segmented in each slice of the axial, sagittal, and coronal views of the PET, CT, and fusion images.

### 2.5 Feature extraction and normalization

A total of 1409 PET and 1409 CT radiomics features were extracted from each segmented ROI using AccuContour software version 3.2 (Manteia Medical Technologies Co. Ltd., Xiamen, China), which is a commercial software application that allows for standardized preprocessing of medical imaging data. The radiomics features based on the original images included shape features, first-order intensity histogram features, gray-level cooccurrence matrix (GLCM) features, gray-level run-length matrix (GLRLM) features, gray-level size zone matrix (GLSZM) features, neighboring gray-tone difference matrices (NGTDM), and gray-level dependence matrix (GLDM) features.

### 2.6 Feature selection and model development

All features were standardized to Z scores with the mean and standard deviation. The Pearson correlation coefficient (PCC) for each feature pair was calculated to evaluate their similarity, and if the PCC value exceeded 0.9, one of the features was randomly eliminated. After this process, the dimension of the feature space was reduced, and features were independent of each other. Then, least absolute shrinkage and selection operator (LASSO) regression analysis with 10-fold cross-validation was employed to select the effective radiomics features. Clinical features were selected using logistic regression analysis. Separate models with good prediction performance were built to differentiate pathological subtypes in locally advanced cervical cancer. Ultimately, the predictive performance of the models was assessed using the receiver operating characteristic (ROC) curve, decision curve analysis (DCA), and calibration curve.

### 2.7 Statistical analysis

Quantitative data that followed a normal distribution are presented as the mean ± standard deviation (s), while qualitative data are expressed as frequencies (percentages). The patient characteristics between the training and validation cohorts were compared using various statistical tests, such as the Pearson Chi-square test, Fisher’s exact test, Student’s t test, and Mann−Whitney U test. Clinical features were selected using univariate and multivariate logistic regression analyses. Six ML classifiers, including logistic regression (LR), naive Bayes (NB), support vector machine (SVM), k-nearest neighbors (KNN), light gradient boosting machine (lightGBM), and multilayer perceptron neural network (MLP), were used to build a model to differentiate pathological subtypes. The optimal ML model was selected based on its AUC, accuracy (ACC), sensitivity (SEN), and specificity (SPE). The AUC values were compared between different models using the DeLong test. The data analyses were performed using SPSS software (Version 25.0, IBM Corp., Armonk, NY, USA) and R software (Version 3.4.0, R Foundation for Statistical Computing, Vienna, Austria). A two-sided p-value<0.05 was considered statistically significant.

## 3 Results

### 3.1 Clinical characteristics and PET metabolic parameters

**Table 1** presents the clinical characteristics and PET metabolic parameters of 227 patients with locally advanced cervical cancer. The comparison between SCC and AC in three groups are shown in **Supplementary Table S1**. The results of the univariate logistic regression analysis are provided in **Table 2**. None of the clinical features or PET metabolic parameters showed significant differentiation ability for the pathological subtypes.

**Table 1:** Comparison of Clinical characteristics and PET metabolic parameters between SCC and AC in the training, internal validation and external validation cohorts.

	Training (N = 136)		*p* Value	Internal validation (N = 59)		*p* Value	External validation (N=32)		*p* Value
	SCC (N=115)	AC (N=21)		SCC (N=45)	AC (N=14)		SCC (N=29)	AC (N=3)	
Age (years)	53.91 ± 9.37	56.33 ± 12.86	0.418	52.67 ± 11.41	55.28 ± 12.22	0.464	52.24 ± 11.27	51.33 ± 4.93	0.892
Abortion						0.105			
NO	64(55.7%)	13(61.9%)	0.595	24(53.33%)	4(28.57%)		11(37.93%)	1(33.33%)	0.876
YES	51(44.3%)	8(38.1%)		21(46.67%)	10(71.43%)		18(62.07%)	2(66.67%)	
MTD (cm)				5.22 ± 1.67		0.29	4.48 ± 1.48	3.27 ± 0.25	0.173
LNM			0.430			0.516			0.909
NO	39(33.9%)	9(42.9%)		13(28.89%)	6(42.86%)		13(44.83%)	2(66.67%)	
YES	76(66.1%)	12(57.1%)		32(71.11%)	8(57.14%)		16(55.17%)	1(33.33%)	
Para-aortic LNM			0.350			0.759			0.476
NO	88(76.5%)	18(85.7%)		37(82.22%)	11(78.57%)		24(82.76%)	2(66.67%)	
YES	27(23.5%)	3(14.3%)		8(17.78%)	3(21.43%)		5(17.24%)	1(33.33%)	
Menopause			0.929			0.849			0.819
NO	45(39.1%)	8(38.1%)		18(40.0%)	6(42.9%)		17(58.62%)	1(33.33%)	
YES	70(60.9%)	13(61.9%)		27(60.0%)	8(57.1%)		12(41.38%)	2(66.67%)	
SUVmax (SUVbw)	15.59 ± 5.88	16.38 ± 8.01	0.672	16.20 ± 5.82	17.27 ± 5.90	0.010	15.30 ± 7.28	9.85 ± 6.81	0.225
MTV (ml)	27.48(15.54,54.08)	34.9(16.85,57.56)	0.555	35.12(22.20,73.87)	27.44(9.21,42.97)	<0.001	11.01(6.10,25.15)	6.56(5.27,13.24)	0.580
SUVmean (SUVbw)	9.23 ± 3.52	9.35 ± 4.43	0.908	10.21 ± 3.44	9.64 ± 2.73	0.013	9.28 ± 4.62	6.08 ± 4.42	0.261
TLG (SUVbw*ml)	231.53(117.85,510.18)	305.64(124.16,693.92)	0.671	405.52(171.37,775.63)	189.45(74.06,294.13)	0.012	101.02(50.60,177.68)	57.04(36.96,64.96)	0.164
WBC count	6.85 ± 2.43	6.56 ± 3.84	0.644	7.54 ± 3.22	6.28 ± 1.91	0.171	6.17 ± 1.85	6.55 ± 0.62	0.730
RBC count	4.12 ± 0.50	4.02 ± 0.43	0.384	4.19 ± 0.65	4.32 ± 0.45	0.494	4.22 ± 0.61	4.45 ± 0.39	0.529
Plt count	291.76 ± 94.11	285.67 ± 101.24	0.788	326.24 ± 103.28	313.57 ± 142.57	0.716	233.25 ± 62.74	225.00 ± 37.51	0.826
lymphocyte count	1.68 ± 0.57	1.56 ± 0.39	0.355	1.63 ± 0.59	1.65 ± 0.40	0.916	3.00 ± 5.79	1.81 ± 0.10	0.728
neutrophile count	4.52 ± 2.16	4.52 ± 3.60	0.995	5.19 ± 2.56	4.01 ± 1.61	0.13	5.57 ± 10.59	4.22 ± 0.66	0.830
Hb count	120.81 ± 16.76	116.71 ± 14.91	0.298	117.98 ± 23.78	116.43 ± 24.42	0.833	126.17 ± 18.74	131.33 ± 1.16	0.642

SCC, squamous cell carcinoma; AC, adenocarcinoma; MTD, maximal tumor diameter; LNM, lymph node metastasis; SUVmax, maximum standardized uptake value; SUVmean, mean standardized uptake value; MTV, metabolic active tumor volume; TLG, total lesion glycolysis; WBC, white blood cell; RBC, red blood cell; WBC, white blood cell; Plt, blood platelet; Hb, hemoglobin.

**Table 2:** Univariate logistic regression analysis of clinical and PET metabolic parameters to differentiate pathological subtypes in the training cohort.

	Univariate logistic analysis		
	OR	95% *CI*	*p*-Value
Age (years)	1.022	0.986-1.060	0.228
Abortion	0.915	0.647-1.295	0.617
MTD (cm)	0.859	0.680-1.087	0.206
LNM	0.642	0.304-1.355	0.245
Para-aortic LNM	0.739	0.284-1.921	0.535
Menopause	0.974	0.462-2.056	0.945
SUVmax (SUVbw)	0.968	0.909-1.031	0.316
MTV (ml)	0.996	0.986-1.006	0.414
SUVmean (SUVbw)	0.933	0.837-1.040	0.211
TLG (SUVbw*ml)	1.000	0.999-1.001	0.385
WBC count	0.911	0.778-1.067	0.249
RBC count	0.995	0.496-1.997	0.989
Plt count	1.000	0.996-1.003	0.806
lymphocyte count	0.780	0.386-1.575	0.488
neutrophile count	0.929	0.780-1.107	0.410
Hb count	0.991	0.972-1.010	0.755

MTD, maximal tumor diameter; LNM, lymph node metastasis; MTV, metabolic active tumor volume; SUV, standardized uptake value; TLG, total lesion glycolysis; WBC, white blood cell; RBC, red blood cell; WBC, white blood cell; Plt, blood platelet; Hb, hemoglobin.

### 3.2 Radiomics features extraction and selection

**Figure 5:**
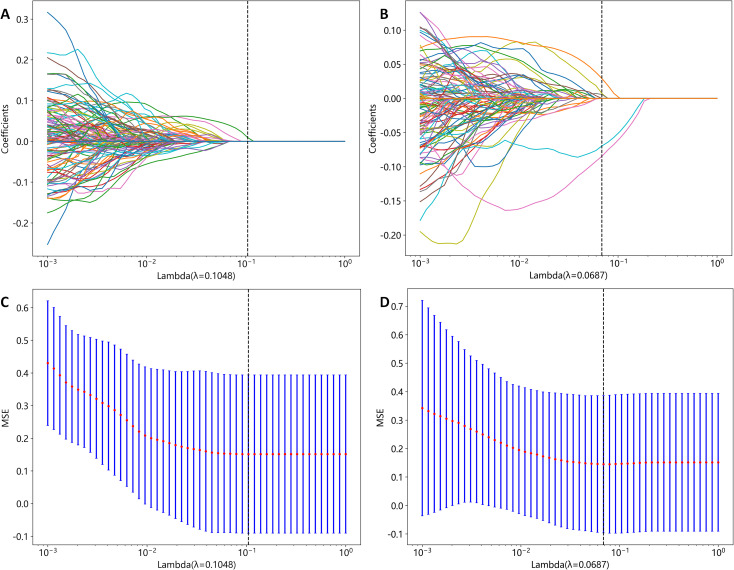
CT and PET radiomics feature selection using the least absolute shrinkage and selection operator (LASSO) algorithm. **(A)** LASSO coefficient profiles of CT radiomics features. **(B)** LASSO coefficient profiles of PET radiomics features. **(C)** Mean square error path obtained through tenfold cross-validation for CT radiomics feature selection process. **(D)** Mean square error path obtained through tenfold cross-validation for PET radiomics feature selection process.

**Figure 6:**
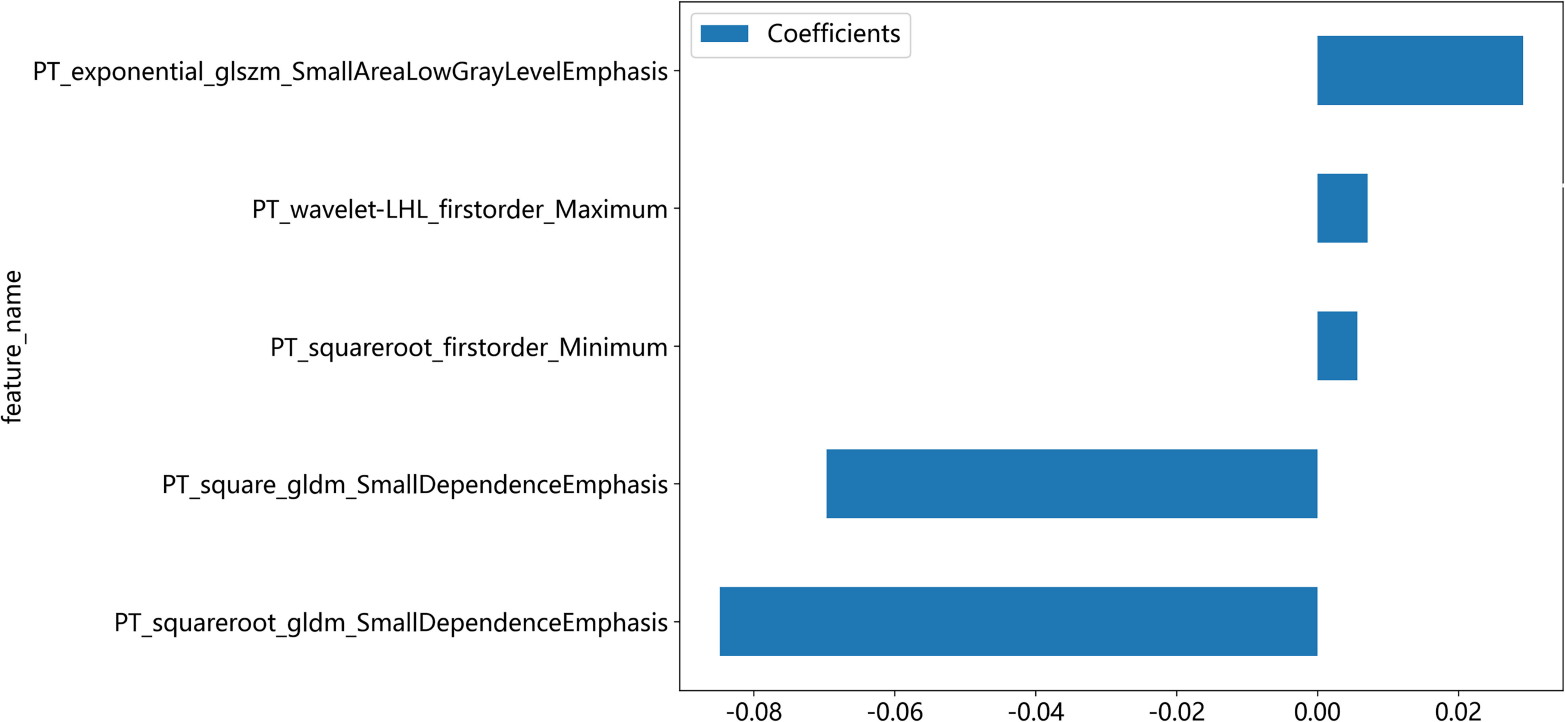
The five PET radiomics features are selected and shown.

A total of 2818 radiomic features were extracted from the ROIs of CT and PET images. Among them, a total of 391 and 242 radiomics features were selected from the CT and PET images, respectively, based on the PCC. Subsequently, LASSO regression analysis was performed to select one CT radiomics feature (**Figures 5A, C**) and five PET radiomic features (**Figures 5B, D**, **6**). Then, **Table 3** displays the final PET and CT radiomic features. The quantitative differences in PET radiomic features between cervical SCC and AC are shown in **Supplementary Table S2**.

**Table 3:** The final PET and CT radiomics features used for models.

Image	Filter	Feature class	Feature
PET	Exponential	GLSZM	Small area low gray level emphasis
	Wavelet(LHL)	First-order	Maximum
	Square	GLDM	Small dependence emphasis
	Squareroot	First-order	Minimum
	Squareroot	GLDM	Small dependence emphasis
CT	Wavelet(HLL)	NGTDM	Busyness

### 3.3 Radiomics model development and evaluation

**Table 4** presents a summary of the prediction performance in distinguishing between AC and SCC using various ML classifiers in the training and internal validation cohorts. The LightGBM model exhibited superior performance in terms of AUC, ACC, SEN, SPE compared to the other ML models, and was consequently employed as the ML algorithm for differentiating the described pathological subtypes.

**Table 4:** Performance of machine learning classifiers for differentiating pathological subtypes in the training and internal validation cohort.

ML	DS	PET radiomics model					CT radiomics model				
		AUC	95% CI	ACC	SEN	SPE	AUC	95% CI	ACC	SEN	SPE
LR	T	0.916	0.852 - 0.979	0.919	0.714	0.957	0.597	0.441 - 0.753	0.779	0.429	0.843
	V	0.779	0.631 - 0.928	0.814	0.571	0.889	0.521	0.330 - 0.711	0.746	0.286	0.909
NB	T	0.848	0.739 - 0.957	0.919	0.667	0.965	0.684	0.549 - 0.820	0.603	0.762	0.574
	V	0.719	0.517 - 0.921	0.847	0.643	0.911	0.524	0.334 - 0.712	0.746	0.286	0.909
SVM	T	0.941	0.885 - 0.998	0.941	0.857	0.957	0.612	0.465 - 0.760	0.632	0.619	0.635
	V	0.811	0.647 - 0.975	0.864	0.786	0.889	0.484	0.287 - 0.681	0.780	0.214	0.977
KNN	T	0.96	0.931 - 0.989	0.824	1.000	0.791	0.802	0.735 - 0.870	0.559	1.000	0.478
	V	0.700	0.535 - 0.865	0.847	0.357	1.000	0.417	0.253 - 0.582	0.763	0.071	1.000
LightGBM	T	0.955	0.922 - 0.988	0.868	0.952	0.852	0.752	0.642 - 0.862	0.713	0.667	0.761
	V	0.851	0.715 - 0.986	0.915	0.643	1.000	0.513	0.339 - 0.688	0.661	0.286	0.814
MLP	T	0.930	0.877 - 0.984	0.809	0.905	0.791	0.597	0.440 - 0.753	0.779	0.429	0.843
	V	0.816	0.667 - 0.965	0.847	0.643	0.911	0.521	0.330 - 0.711	0.746	0.286	0.909

ML, machine learning; DS, data set; PET, positron emission tomography; CT, computed tomography; AUC, area under the curve; CI, confidence interval; ACC, Accuracy; SEN, Sensitivity; SPE, Specificity; LR, logistic regression; T, training cohort; V, internal validation cohort; NB, Naive Bayes; SVM, support vector machine; KNN, k-nearest neighbors; lightGBM, light gradient boosting machine; MLP, multilayer perceptron neural network.

**Figure 7:**
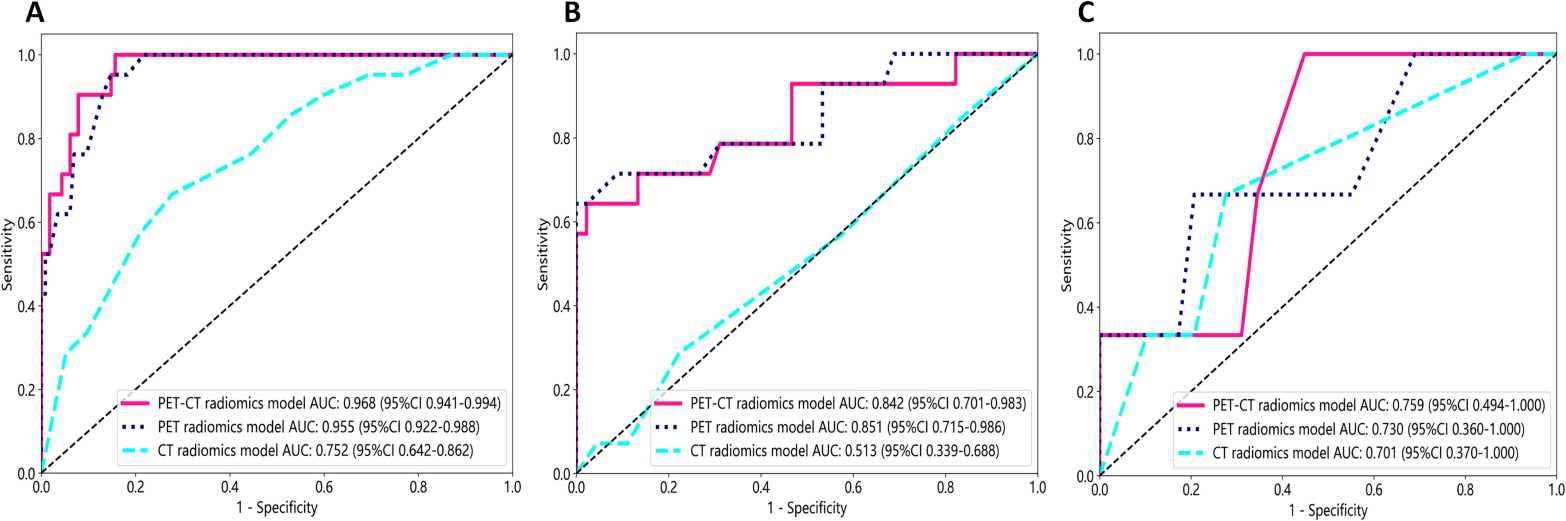
The receiver operating characteristic (ROC) curves of all three radiomics models were used to differentiate pathological subtypes in the training cohort **(A)**, internal validation cohort **(B)**, and external validation cohort **(C)**.

**Figure 7** illustrates the ROC curves of the CT radiomics model, PET radiomics model, and combined model. In the training cohort, the best differentiation performance was demonstrated by the combined radiomics model (AUC=0.968), followed by the PET radiomics model (AUC=0.955), while the differentiation performance of the CT radiomics model was average (AUC=0.752). The DeLong test indicated that there was no statistically significant difference between the combined radiomics model and the PET radiomics model (*z*=0.940, p-value=0.347). Nevertheless, both the combined radiomics model and the PET radiomics model significantly outperformed the CT radiomics model (*z*=3.291, p-value<0.001). In the internal validation cohort, the PET radiomics model had the best differentiation effectiveness (AUC=0.851), followed by the combined radiomics model (AUC=0.842), while the differentiation performance of the CT radiomics model was poor (AUC=0.513). The DeLong test showed no statistically significant difference between the combined radiomics model and the PET radiomics model (*z*=0.285, p-value=0.776). However, both the combined radiomics model and the PET radiomics model were significantly better than the CT radiomics model (*z*=2.807, p-value=0.005 and *z*=2.697, p-value=0.007, respectively). In the external validation cohort, the DeLong test showed no statistically significant difference between the combined radiomics model and the PET radiomics model (*z*=0.272, p-value=0.809).

The DCA results showed that the PET radiomics model performed better and provided a higher clinical application value in differentiating pathological subtypes than CT radiomics mode and PET-CT radiomics model (**Supplementary Figure S1**). The calibration curves for the training cohort, internal validation cohort and external validation cohort (**Supplementary Figures S2**), assessed using the Hosmer-Lemeshow test, showed no significant differences in both the training cohort (P=0.129), internal validation cohort (P=0.351) and external validation cohort (P=0.529). This suggests good consistency between the actual and predicted risks.

## 4 Discussion

In this study, we successfully developed six ML models based on PET and CT images, among which the lightGBM model based on PET radiomics features performed excellently in distinguishing AC and SCC.

Previous literature has indicated that CT radiomics features exhibit better predictive performance than PET radiomics features in predicting survival, and CT radiomics features are also more abundant than PET features (22, 23). Nevertheless, with regard to distinguishing pathological subtypes, Kirienko et al. (24) discovered that PET radiomics features had a greater ability to discriminate between primary and metastatic pulmonary lesions than CT radiomics features. Further studies conducted by Hyun et al. (25) and Han et al. (21) demonstrated that a PET/CT-based machine learning method was able to make a distinction between AC and SCC in patients with non-small cell lung cancer. This study indicates that in distinguishing SCC and AC, the selected PET radiomics features are substantially more numerous than CT radiomics features, and the performance of the PET radiomics model is notably superior to that of the CT radiomics model. Furthermore, the Delong test showed that although there was a slight improvement in performance when combining PET radiomics features with CT radiomics features, the increase in AUC value did not reach statistical significance (the p values of the training and internal validation cohorts were 0.347 and 0.776, respectively). In a retrospective study, Shen et al. (19) first found that short-zone emphasis (SZE) from GLSZM was the only PET-based radiomics feature that showed quantitative differences between SCC and non-SCC in cervical cancer. Tsujikawa et al. (20) reported that the correlation from normalized gray-level co-occurrence matrix (NGLCM) was the only feature extracted from ^18^F-FDG PET that showed significant differences between cervical SCC and non-SCC. The previous two published studies extracted merely 18 or 76 features from the original images. In contrast, our study extracted 2818 features from the original images as well as the converted images. This might be the cause for which the previous study could select only one meaningful feature, while our study selected five. In summary, the findings of this series of PET/CT radiomic studies highlights the importance of functional imaging-based radiomics research in differentiating tumor pathological subtypes. This may be related to the FDG uptake heterogeneity between AC and SCC, which is consistent with the identification of the pathological subtypes of lung cancer based on PET/CT (21).

MRI techniques also introduced various functional sequences, including apparent diffusion coefficient (ADC), dynamic contrast-enhanced imaging, and perfusion-weighted imaging (26). Wang et al. (18) achieved good differentiation between SCC and AC using a multiparameter MRI radiomics model based on ADC, enhanced T1-weighted imaging, and other anatomical and functional sequences. Although the differentiation performance of the multi-parametric MRI-based radiomics model was the highest among the published MRI-based radiomics studies, its differentiation performance (AUC = 0.89) was lower than that of the pure PET-based radiomics model constructed in our study (AUC = 0.955). These findings demonstrate the advantages of PET radiomics features over multiparametric MRI radiomics features to a certain extent. PET-based radiomics can not only reveal the intratumoral heterogeneity of imaging structures between AC and SCC but also demonstrate the heterogeneity of tumor cell metabolism. Moreover, radiomics features are based on manually segmented ROIs in five MRI sequences, which not only requires a substantial amount of work but also increases the instability of the features.

The radiomics features selected in our study are all derived from processed images, which may reveal greater tumor heterogeneity differences between SCC and AC compared to the original images, showcasing the advantage of radiomics. Among these radiomics features selected in our study, the firstorder_Maximum and firstorder_Minimum represent the maximum and minimum gray level intensities, respectively. SCC exhibits significantly higher values than AC, indicating that SCC has a stronger FDG uptake than AC. Campos-Parra et al. found that compared to AC, SCC exhibits higher activation levels of key cancer pathways, such as IL-17, JAK/STAT, and Ras signaling (27). high-risk human papilloma virus (HPV) -16 infection is more common in SCC, while HPV-18 and HPV-45 are more frequently observed in AC (27, 28). Priego-Hernández et al. discovered that cervical cancer and HPV-16-positive cell lines have increased expression of HIF-1α and glucose metabolism-related genes (GLUT1, LDHA, CAIX, MCT4, and BSG) (29). Furthermore, there are significant variations in the expression of glucose metabolism-related genes between SCC and AC (30). Choi et al. demonstrated that tumor FDG uptake is associated with glucose transporters (Glut-1 and Glut-3), with SCC exhibiting higher expression intensity and proportion of Glut-1 compared to AC. Consequently, SCC demonstrates higher SUVmax and stronger FDG uptake capacity (31). Small dependence emphasis (SDE) from GLDM and small area low gray level emphasis (SALGLE) from GLSZM represent tumor heterogeneity, with higher values indicating more significant heterogeneity. In our study, GLDM_SDE and GLSZM_SALGLE features were significantly higher in SCC compared to AC, indicating that the intratumoral metabolic heterogeneity based on PET imaging in SCC is significantly higher than that in AC. This may be related to the previously mentioned metabolic and histomorphological differences between SCC and AC. The tissue structure of SCC is tight, with small gaps between tumor cells, wrapped in several matrix structures, forming cancer nests. In contrast, the tissue structure of AC is more loose, characterized by glandular differentiation. Therefore, the differential expression of pathogenic molecular mechanisms, especially glucose metabolism genes, determines the metabolic differences of tumor cells, while cell arrangement and tissue morphology determine the spatial heterogeneity of tumor cells. The tumor heterogeneity revealed by PET images manifests these metabolic differences and spatial heterogeneity of tumor cells. These findings require further validation with a larger-scale patient or in combination with pathomics.

In this study, we employed six ML algorithms to develop models for distinguishing SCC and AC. Among the algorithms, the radiomics model constructed by the LightGBM algorithm exhibited excellent differentiation performance, accuracy, sensitivity, and specificity with a relatively balanced performance. This finding is consistent with a similar study conducted by Lam et al., who investigated the correlation between radiomics features and tumor mutation burden in glioma based on MRI images using LR, SVM, and six other ML algorithms (32). They found that the radiomics model constructed by the LightGBM algorithm also demonstrated the best discriminative performance with relatively balanced sensitivity and specificity. Furthermore, researchers have successfully achieved good discriminative performance in distinguishing low-grade and high-grade meningiomas using the LightGBM algorithm for both radiomics and deep learning models (33). Similarly, Chang et al. constructed LightGBM and convolutional neural network (CNN) models based on noncontrast CT and enhanced images to differentiate thymic epithelial tumors from other anterior mediastinal tumors (34). The results demonstrated that the LightGBM model outperformed the CNN model in both the noncontrast CT dataset and the enhanced CT dataset. The LightGBM algorithm, which is based on the gradient boosting decision tree model, optimizes the search for optimal split points and the tree growth process. It supports efficient parallel training and possesses advantages such as faster training speed, lower memory consumption, better accuracy, and quick processing of massive data, making it widely applicable. Therefore, ML can better handle complex nonlinear relationships in large-scale datasets and holds great potential for clinical applications (35). However, it is important to acknowledge that ML models and algorithms also have limitations, including overfitting and lack of interpretability. Overfitting can undermine predictive performance, while the lack of interpretability can hinder the use of ML (36). Hence, it is essential to prioritize the future optimization of ML algorithms and conduct independent validations to verify their performance.

There were several limitations in this study. Firstly, it was a retrospective and preliminary study, carrying a potential selection bias despite the use of strict inclusion and exclusion criteria. Secondly, HPV status and histological differentiation were not available for some patients when retrieving the electronic medical record system, and we were unable to further explore their impact on pathological subtypes. Lastly, the sample size of AC in this study is relatively small, but this is consistent with the epidemiology of cervical cancer. To improve the generalizability of the model, it is necessary to investigate a larger sample size from multiple centers in future research.

## 5 Conclusion

The lightGBM-based PET radiomics model effectively identified pathological subtypes in patients with locally advanced cervical cancer and may help clinicians in their daily decision-making process.

## Data Availability

The original contributions presented in the study are included in the article/**Supplementary Material**. Further inquiries can be directed to the corresponding author.
