# Supplementary material for: Radiomics-based machine learning models for differentiating pathological subtypes in cervical cancer: a multicenter study
Source: Front Oncol. 2024 Sep 17;14:1346336. doi: 10.3389/fonc.2024.1346336 (PMC11442173; doi:10.3389/fonc.2024.1346336)
Supplement: Supplementary file 1 [file DataSheet1.docx]

**TABLE S1.** Clinical characteristics and PET metabolic parameters in the training, interval validation and external validation cohorts

|  | Training  (N = 136) | Internal validation  (N = 59) | *t*/χ2/Z | *p* Value | External validation  (N=32) |
| --- | --- | --- | --- | --- | --- |
| Age (years) | 54.29±9.97 | 53.29±11.55 | 0.612^a^ | 0.541 | 52.03±10.79 |
| Pathology |  |  | 1.919^b^ | 0.166 |  |
| SCC | 115(84.6%) | 45(76.3%) |  |  | 29(90.63%) |
| AC | 21(15.4%) | 14(23.7%) |  |  | 3(9.37%) |
| Abortion |  |  | 1.389^b^ | 0.239 |  |
| NO | 77(56.6%) | 28(47.5%) |  |  | 13(40.63%) |
| YES | 59(43.3%) | 31(52.5%) |  |  | 19(59.37%) |
| MTD (cm) | 4.96±1.62 | 5.22±1.67 | -1.062^a^ | 0.290 | 4.35±1.46 |
| LNM |  |  | 0.174^b^ | 0.676 |  |
| NO | 48(35.3%) | 19(32.20%) |  |  | 15(46.88%) |
| YES | 88(64.7%) | 40(67.80%) |  |  | 17(53.12%) |
| Para-aortic LNM |  |  | 0.289^b^ | 0.591 |  |
| NO | 106(77.9%) | 48(81.4%) |  |  | 26(81.25%) |
| YES | 30(22.1%) | 11(18.6%) |  |  | 6(18.75%) |
| Menopause |  |  | 0.050^b^ | 0.823 |  |
| NO | 53(39.0%) | 24(40.7%) |  |  | 18(56.25%) |
| YES | 83(61.0%) | 35(59.3%) |  |  | 14(43.75%) |
| SUVmax (SUVbw) | 15.72±6.23 | 16.20±5.82 | -0.503^a^ | 0.615 | 14.79±7.31 |
| MTV (ml) | 29.43(15.53,54.09) | 33.42(21.42, 59.93) | -1.202^c^ | 0.230 | 10.22(5.51, 22.53) |
| SUVmean (SUVbw) | 9.25±3.66 | 9.59±3.44 | -0.613^a^ | 0.540 | 8.98±4.63 |
| TLG (SUVbw*ml) | 253.07(119.97,552.82) | 331.91(154.36, 662.90) | -1.061^c^ | 0.289 | 84.38(43.17, 174.93) |
| WBC count | 6.81±2.68 | 7.24±2.99 | 0.236^a^ | 0.316 | 6.20±1.77 |
| RBC count | 4.11±0.49 | 4.22±0.61 | -1.378^a^ | 0.170 | 4.21±0.55 |
| Plt count | 290.82±99.88 | 323.24±109.59 | -1.934^a^ | 0.056 | 234.57±58.80 |
| lymphocyte count | 1.66±0.54 | 1.63±0.55 | 0.275^a^ | 0.783 | 2.86±5.52 |
| neutrophile count | 4.51±2.42 | 4.92±2.41 | -1.069^a^ | 0.286 | 5.47±10.06 |
| Hb count | 120.18±16.51 | 117.61±23.73 | 0.755^a^ | 0.452 | 126.63±17.90 |

SCC, squamous cell carcinoma; AC, adenocarcinoma; MTD, maximal tumor diameter; LNM, lymph node metastasis; SUVmax, maximum standardized uptake value; SUVmean, mean standardized uptake value; MTV, metabolic active tumor volume; TLG, total lesion glycolysis; WBC, white blood cell; RBC, red blood cell; WBC, white blood cell; Plt, blood platelet; Hb, hemoglobin; ^a^ t value; ^b^ χ2 value ; ^c^ Z value;

**TABLE S2.** Comparison of PET radiomics features in the training, interval validation and external validation cohorts

| PET radiomics features | Training cohort | | |  | Internal validation cohort | | |  | External validation cohort | | |
| --- | --- | --- | --- | --- | --- | --- | --- | --- | --- | --- | --- |
|  | SCC | AC | P |  | SCC | AC | P |  | SCC | AC | P |
| wavelet_LHL_firstorder_Maximum | 6648.45 （3111.57, 8492.56） | 4770.46 （3665.32, 6552.02） | 0.027 |  | 5324.82 （3751.60, 6825.62） | 4113.67 （2017.957, 6719.66） | 0.018 |  | 5336.81 （3722.82, 7543.16） | 3201.51 （2328.23, 3681.56） | 0.028 |
| square_GLDM_SDE | 0.96 （0.94, 0.97） | 0.8 （0.65, 0.96） | ＜0.001 |  | 0.96（0.93, 0.98） | 0.76 （0.52, 0.96） | 0.001 |  | 0.98 （0.95, 1.00） | 0.95 （0.85, 0.98） | 0.017 |
| squareroot_firstorder_  Minimum | 25357.1 （19567.76, 31498.91） | 20057.94 （15934.90, 27525.00） | 0.039 |  | 20404.22 （17060.60, 29384.67） | 16157.53 （12796.64, 23305.43） | 0.019 |  | 27651.30 （22681.53, 40145.75） | 19808.66 （17507.34, 32103.74） | 0.041 |
| squareroot_GLDM_SDE | 0.96 （0.94, 0.97） | 0.95 （0.74, 0.97） | 0.047 |  | 0.96 （0.94, 0.98） | 0.75 （0.67, 0.95） | ＜0.001 |  | 0.98 （0.96, 0.99） | 0.95 （0.95, 0.98） | 0.062 |
| exponential_GLSZM_  SALGLE | 0.02 （0.01, 0.03） | 0.03 （0.02, 0.07） | 0.020 |  | 0.02 （0.01, 0.04） | 0.02 （0.01, 0.10） | 0.036 |  | 0.05 （0.04, 0.07） | 0.08 （0.04, 0.13） | 0.058 |

PET, positron emission tomography; SCC, squamous cell carcinoma; AC, adenocarcinoma; SDE, small dependence emphasis; GLDM, gray-level dependence matrix; SDE, small dependence emphasis; GLSZM, gray-level size zone matrix; SALGLE, small area low gray level emphasis


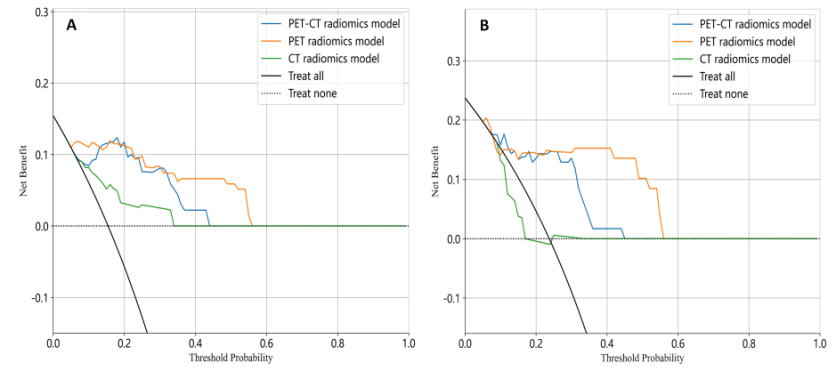

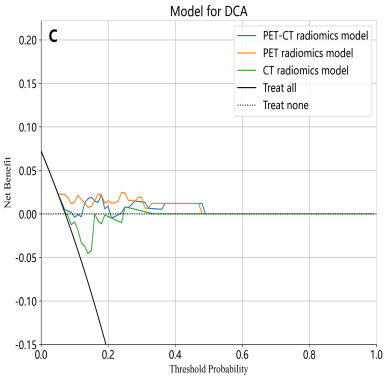


**Figure S1.** The decision curve analysis (DCA) curves. The decision curves show that the PET radiomics model performed better and provided a higher clinical application value than CT radiomics mode and PET-CT radiomics model in training cohort (A), internal validation cohort (B) and external validation cohort (C).


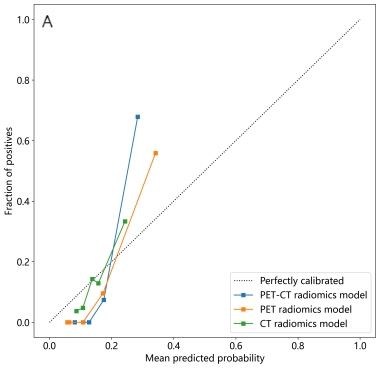

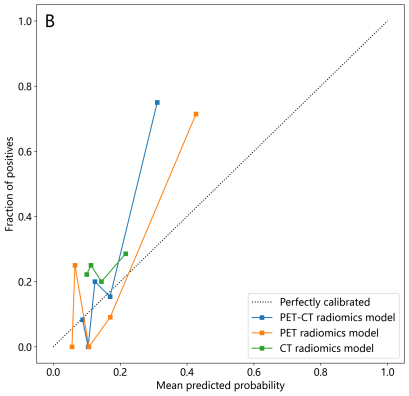

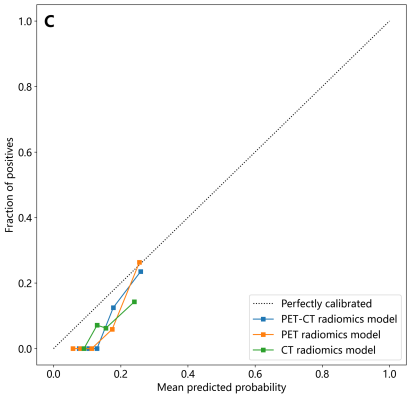


**Figure S2.** Calibration curves of the models in training cohort (A), internal validation cohort (B) and external validation cohort (C).
